# Control of Plant Trichome and Root-Hair Development by a Tomato (*Solanum lycopersicum*) R3 MYB Transcription Factor

**DOI:** 10.1371/journal.pone.0054019

**Published:** 2013-01-11

**Authors:** Rumi Tominaga-Wada, Yuka Nukumizu, Shusei Sato, Takuji Wada

**Affiliations:** 1 Interdisciplinary Research Organization, University of Miyazaki, Miyazaki, Japan; 2 Kazusa DNA Research Institute, Chiba, Japan; Wake Forest University, United States of America

## Abstract

In *Arabidopsis thaliana* the CPC-like MYB transcription factors [CAPRICE (CPC), TRIPTYCHON (TRY), ENHANCER OF TRY AND CPC 1, 2, 3/CPC-LIKE MYB 3 (ETC1, ETC2, ETC3/CPL3), TRICHOMELESS 1, 2/CPC-LIKE MYB 4 (TCL1, TCL2/CPL4)] and the bHLH transcription factors [GLABRA3 (GL3) and ENHANCER OF GLABRA 3 (EGL3)] are central regulators of trichome and root-hair development. We identified *TRY* and *GL3* homologous genes from the tomato genome and named them *SlTRY* and *SlGL3,* respectively. Phylogenic analyses revealed a close relationship between the tomato and Arabidopsis genes. Real-time reverse transcription PCR analyses showed that *SlTRY* and *SlGL3* were predominantly expressed in aerial parts of developing tomato. After transformation into Arabidopsis, *CPC::SlTRY* inhibited trichome formation and enhanced root-hair differentiation by strongly repressing *GL2* expression. On the other hand, *GL3::SlGL3* transformation did not show any obvious effect on trichome or non-hair cell differentiation. These results suggest that tomato and Arabidopsis partially use similar transcription factors for epidermal cell differentiation, and that a CPC-like R3 MYB may be a key common regulator of plant trichome and root-hair development.

## Introduction

Epidermal cell differentiation, including trichome and root-hair formation, in *Arabidopsis thaliana* is a popular model system for studying cell fate determination. Several regulatory factors are known to be involved in this event. The *CAPRICE* (*CPC*) gene encodes an R3 type MYB transcription factor that has been identified as a key regulator of root-hair differentiation [1]. Arabidopsis has several additional *CPC*-like MYB genes in its genome, including *TRYPTICHON* (*TRY*), *ENHANCER OF TRY AND CPC1* and *2* (*ETC1* and *ETC2*), *ENHANCER OF TRY AND CPC3/CPC-LIKE MYB3* (*ETC3/CPL3*), and *TRICHOMELESS1* and *2/CPC-LIKE MYB4* (*TCL1* and *TCL2/CPL4*) [2]–[10]. The TRY protein has a regulatory role mainly in trichome differentiation [2], [11]. ETC1 and ETC2 enhance the functions of CPC and TRY [3]–[5]. TCL1 and TCL2/CPL4 negatively regulate trichome formation on the inflorescence stems and pedicels [8]–[10].

The *GLABRA3* (*GL3*) gene encodes a bHLH transcription factor that is also involved in trichome and root-hair differentiation in Arabidopsis [12]. A *GL3* homologous gene, *ENHANCER OF GLABRA3* (*EGL3*), functions in a redundant manner with *GL3* in Arabidopsis [13]. The *GLABRA2* (*GL2*) gene, which encodes a homeodomain leucine zipper protein, is thought to act farthest downstream in the epidermal cell fate regulatory pathway in Arabidopsis [1], [14]–[17]. Transcription of *GL2* is controlled by a protein complex that includes the WEREWOLF (WER), GL3/EGL3 and TRANSPARENT TESTA GLABRA1 (TTG1) proteins [18]. WER encodes an R2R3 type MYB transcription factor and promotes the differentiation of Arabidopsis root epidermal cells into non-hair cells [16]. The *TTG1* gene, which encodes a WD-40 protein, is also required for the formation of non-hair cells [14]. Two bHLH proteins, GL3 and EGL3, interact with WER [13] and TTG1 [12], [19], [20]. The *WER* homologous gene *GLABRA1* (*GL1*) is also thought to form a transcriptional complex with GL3/EGL3 and TTG1 to promote *GL2* expression [12], [20]–[22]. The CPC and CPC-like MYB proteins interact with GL3/EGL3 and may serve as epidermal cell fate determinants [7].

Although both tomato and Arabidopsis have trichomes, tomato trichomes are distinct from Arabidopsis trichomes. Arabidopsis has non-glandular three-branched unicellular trichomes that form on stem and leaf surfaces to an extent that depends on the ecotype [23], [24]. On the other hand, tomato trichomes are highly diverse in morphology and chemistry [25]–[27]. Tomato trichomes are classified into types I–VII, with types I, IV, VI and VII being glandular, and types II, III and V being non-glandular [26], [28]. Glandular trichomes contain various sticky or toxic chemicals that may resist herbivores [27], whereas non-glandular trichomes may function in defense by physically limiting herbivores [29].

Trichome morphology and root-hair patterning are different in tomato and Arabidopsis. Arabidopsis root-hair cells are located over two underlying cortical cells, whereas non-hair cells are positioned over a single cortical cell [14], [30]. This position-dependent pattern results in rows of root-hair cells along the longitudinal root axis and has been found in Brassicaceae and other eudicot families [31]–[34]. This striped root-hair pattern (Type 3) is one of three types of root-hair cell distribution patterns [31]–[33], [35]–[38]. Tomato belongs to the Type 1 root-hair pattern group, in which all the root epidermal cells have the potential to produce root-hairs. This type of pattern appears to be the most widespread in plants [39].

In this study, we have identified Arabidopsis *TRY* and *GL3* homologous genes from tomato. Transformants expressing the tomato *TRY* homologous gene (*SlTRY*) in Arabidopsis had no trichomes and a greater number of root-hairs, a phenotype similar to that seen in over-expressors of *CPC*-like MYB genes. On the other hand, transformants expressing the tomato *GL3* homologous gene (*SlGL3*) in Arabidopsis had no obvious *GL3*-like effects on trichome and non-hair cell differentiation. We concluded that tomato and Arabidopsis use similar transcription factors for trichome and root-hair cell differentiation and that the *SlTRY*-like R3 MYB may be a key common regulator of plant trichome and root-hair development.

## Materials and Methods

### Plant Materials and Growth Conditions

Tomato, *Solanum lycopersicum* L. cv. Micro-Tom, was used. Seeds were surface-sterilized with 10% commercial bleach including a detergent (Kitchen Haiter, Kao, Tokyo, Japan), for 20 min and then rinsed with sterilized water three times for 5 min each and sown on 1.5% agar plates containing 0.5xMS medium [40]. Seeded plates were kept at 4°C for 2 d and then incubated at 25°C under constant white light (50–100 µmol m^−2^ s^−1^) for 7 days to produce seedlings for DNA and RNA extraction. Some 7-day-old seedlings were transplanted into soil and grown in a photoperiod of 16 h light at 25°C for 4 additional weeks to produce mature plant tissues for RNA extraction.

*Arabidopsis thaliana* ecotype Columbia (Col-0) and, cognate *cpc-2* [41] and *gl3-7454* [42] mutant plants were used. Seeds were surface-sterilized, sown on 1.5% agar plates as described previously [43] and propagated to observe seedling phenotypes. Seeded plates were kept at 4°C for 2 d and then incubated at 22°C under constant white light (50–100 µmol m^−2^ s^−1^). For each transgenic line, at least ten individual 5-day-old seedlings were assayed for root-hair number, and at least five individual 2-week-old third leaves were assayed for trichome number.

### Gene Constructs

#### Primers

All primer sequences used in this paper are listed in Table 1.

**Table 1:** Primer sequences used in this study.

Primer Name	Sequence (5′ to 3′)
RTSlTRY-F	5′-CGATGTTGCAGCCAATGAAGA-3′
RTSlTRY-R	5′-TGTGCAAACCCATCACTGTGTC-3′
RTSlGL3-F	5′-AATGTTGGCCAAGGGTTACCAG-3′
RTSlGL3-R	5′-AAAGACTTTACTCTCGGCTTGGTGA-3′
LeActin-F	5′-TGTCCCTATTTACGAGGGTTATGC-3′
LeActin-R	5′-CAGTTAAATCACGACCAGCAAGAT-3′
GL2-F	5′-ATCGTCACACCACCGATCAGA-3′
GL2-R	5′-CCAGCCCTAGTTGCTTGCTCA-3′
GFP-F	5′-CAGTCCGCCCTGAGCAAAGAC-3′
GFP-R	5′-CCCTTGCTCACCATGGACTTGTA-3′
Act2-F	5′-CTGGATCGGTGGTTCCATTC-3′
Act2-R	5′-CCTGGACCTGCCTCATCATAC-3′
SlTRY-F01	5′-TGAAACCGGTCTCGAGATGTGGTTAAGC-3′
SlTRY-R01	5′-TTTGATCCATCGAACTAATCTGAAGACACG-3′
SlTRY-F02	5′-GATTAGTTCGATGGATCAAAATCTCCATCAC-3′
SlTRY-R02	5′-CGGCGGCTGTAGGTGGTAGACTTTTCTTAATTG-3′
SlTRY-F03	5′-TCTACCACCTACAGCCGCCGCCGCCATGGTGAG -3′
SlTRY-R03	5′-ACGAATTCGAGCTCGGTACCCGGGGATCCTC-3′
SlGL3-F01	5′-GGGGGAACTCCTCGAGGCCAAAC-3′
SlGL3-R01	5′-CCATAGCCATTGTTTCTTCATCCCTATATC-3′
SlGL3-F02	5′-TGAAGAAACAATGGCTATGGGACACCAAG-3′
SlGL3-R02	5′-GGTGGATGGGAGATTTCCATACTACTCTCTG-3′
SlGL3-F03	5′-ATGGAAATCTCCCATCCACCATTTACGAACG-3′
SlGL3-R03	5′-ACGAATTCGAGCTCGGTACC-3′
SlTRY-P2	5′-CAAATGTTTGAACGATCTGC-3′
SlTRY-P3	5′-GAATGAACTTGTTGGCCCTAC-3′
SlTRY-P4	5′-CATAGAAGGGACATACTGGT-3′
SlTRY-VP1	5′-GTATACAACAAATGTGCTTC-3′
SlTRY-VP2	5′-GAGTTAGCTCACTCATTAGG-3′
SlGL3-P1	5′-CTAATGGTATTCTAGTCAAC-3′
SlGL3-P4	5′-CACTGACTGACCTACATATG-3′
SlGL3-P5	5′-CACCTTGAACCCGTCTATTG-3′
SlGL3-VP1	5′-CTATAGGGAGAATCAACGTC-3′
SlGL3-VP2	5′-CAATTAATGTGAGTTAGCTC-3′
SlGL3-F1	5′-TCAGGCGGGGGAAGTTAATG-3′
SlGL3-F2	5′-AATCCTCTTTGCCTCACCAG-3′
SlGL3-F3	5′-ATTAACTTTGGGACCACATT-3′
SlGL3-R1	5′-AAATTACCCTTGGCCAACAT-3′
SlGL3-R2	5′-TCCATTTGACATATTTTAGG-3′
SlGL3-R3	5′-GTCATCAACTTCTGGTCTCC-3′

### *CPC::SlTRY* Construct

A 1.0-kb PCR-amplified linear *CPC* promoter sequence (primers SlTRY-F01/R01) from the Arabidopsis genome, a 0.8-kb PCR-amplified linear *SlTRY* tomato genomic fragment (primers SlTRY-F02/R02) and a 1.8-kb PCR-amplified 2xGFP fragment [41] (primers SlTRY-F03/R03) using PrimeSTAR HS DNA Polymerase and TaKaRa LA Taq (Takara, Tokyo, Japan) were ligated into the *Xho*I and *Kpn*I sites of *pJHA212K* binary vector [44] using an In-Fusion HD Cloning Kit (Takara, Tokyo, Japan) to create *CPC::SlTRY*. PCR-generated constructs were completely sequenced following isolation of the clones to check for amplification-induced errors. The plasmid of *CPC::SlTRY* was sequenced using the SlTRY-P2, -P3, -P4, -F02, -F03, -VP1 and -VP2 primers.

### *GL3::SlGL3* Construct

A 1.3-kb PCR-amplified linear *GL3* promoter sequence (primers SlGL3-F01/R01) from the Arabidopsis genome, a 4.3-kb PCR-amplified linear *SlGL3* tomato genomic fragment (primers SlGL3-F02/R02) and a 1.0-kb PCR-amplified GFP fragment [41] (primers SlGL3-F03/R03) using PrimeSTAR HS DNA Polymerase and PrimeSTAR GXL DNA Polymerase (Takara, Tokyo, Japan) were ligated into the *Xho*I and *Kpn*I sites of *pJHA212K* binary vector [44] using an In-Fusion HD Cloning Kit (Takara, Tokyo, Japan) to create *CPC::SlGL3*. PCR-generated constructs were completely sequenced following isolation of the clones to check for amplification-induced errors. The plasmid of *CPC::SlGL3* was sequenced using the SlGL3-P1, -P4, -P5, -F1, -F2, -F3, -F03, -R1, -R2, -R3, -VP1 and -VP2 primers.

### Transgenic Plants

Gene constructs were introduced into *Agrobacterium tumefaciens* C58C1. Arabidopsis plants (wild-type Col-0, *cpc-2*, and *gl3-7454*) were transformed by the floral dipping method [45] and screened on 0.8% agar plates containing diluted (50% v/v) Murashige and Skoog medium and 50 mg/L (for Col-0, and *gl3-7454* background) or 100 mg/L (for *cpc-2* background) kanamycin sulfate. Homozygous transgenic lines were selected based on kanamycin resistance. We isolated at least twenty T1 lines for each construct and selected at least ten T2 and five T3 lines on the basis of their segregation ratios for kanamycin resistance.

### Real-time Reverse Transcription PCR Analysis

Total RNA from tomato or Arabidopsis tissues was extracted with MagDEA RNA 100 (GC) (PSS, Chiba, Japan) using a Magtration System 12 GC (PSS, Chiba, Japan). To remove contaminating genomic DNA, RNA samples were treated with DNase I (Ambion, Austin, TX, USA) according to the Magtraction System protocol. Plant tissue (100 mg) was homogenized using a TissueLyser II (Qiagen, Valencia, CA, USA) with 100 µl of RLT buffer (Qiagen, Valencia, CA, USA). Sample supernatants were applied to the instrument, and RNA was eluted with 50 ml of sterile distilled water.

First-strand cDNA was synthesized from 1 µg total RNA in a 20 µl reaction mixture using the Prime Script RT Master Mix (Perfect Real Time) (Takara, Tokyo, Japan). Real-time PCR was performed using a Chromo4 Real-Time IQ5 PCR Detection System (Bio-Rad, Hercules, CA, USA) with SYBR Premix Ex Taq II (Takara, Tokyo, Japan). PCR amplification employed a 30 s denaturing step at 95°C, followed by 5 s at 95°C and 30 s at 60°C with 40 cycles for *SlTRY, SlGL3, LeActin, GL2, GFP* and *ACT2*. Real-time PCR was used to analyze the mRNA expression level of each transcript encoding *SlTRY* and *SlGL3* in tomato, and *GL2* and *GFP* in *Arabidopsis* transformants. The relative expression of each transcript was calculated by the ΔΔCT method [46]. The expression levels of *SlTRY* and *SlGL3* were estimated after being normalized to the endogenous control gene *LeActin* (TC116322). The expression levels of *GL2* and *GFP* were estimated after being normalized to the endogenous control gene *ACT2* (AB026654). The primers were: *RTSlTRY-F* and *RTSlTRY-R* for *SlTRY*; *RTSlGL3-F* and *RTSlGL3-R* for *SlGL3*; *LeActin-F* and *LeActin-R* for *LeActin* [47]; *GL2-F* and *GL2-R* for *GL2* [48]; *GFP-F* and *GFP-R* for *GFP*; and *Act2-F* and *Act2-R* for *ACT2* [49].

### Light Microscopy

To observe trichomes, images were recorded with a VC4500 3D digital fine microscope (Omron, Kyoto, Japan) or a digital microscope (VH-8000; Keyence, Osaka, Japan). At least five 2-week-old third true leaves were analyzed for trichome number for each transgenic line. Root phenotypes were observed using an Olympus Previs AX70 microscope and an Olympus SZH binocular microscope. For each transgenic line, at least ten individual 5-day-old seedlings were analyzed for root-hair number.

## Results

### Identification of the *SlTRY* and *SlGL3* Genes

**Figure 1:**
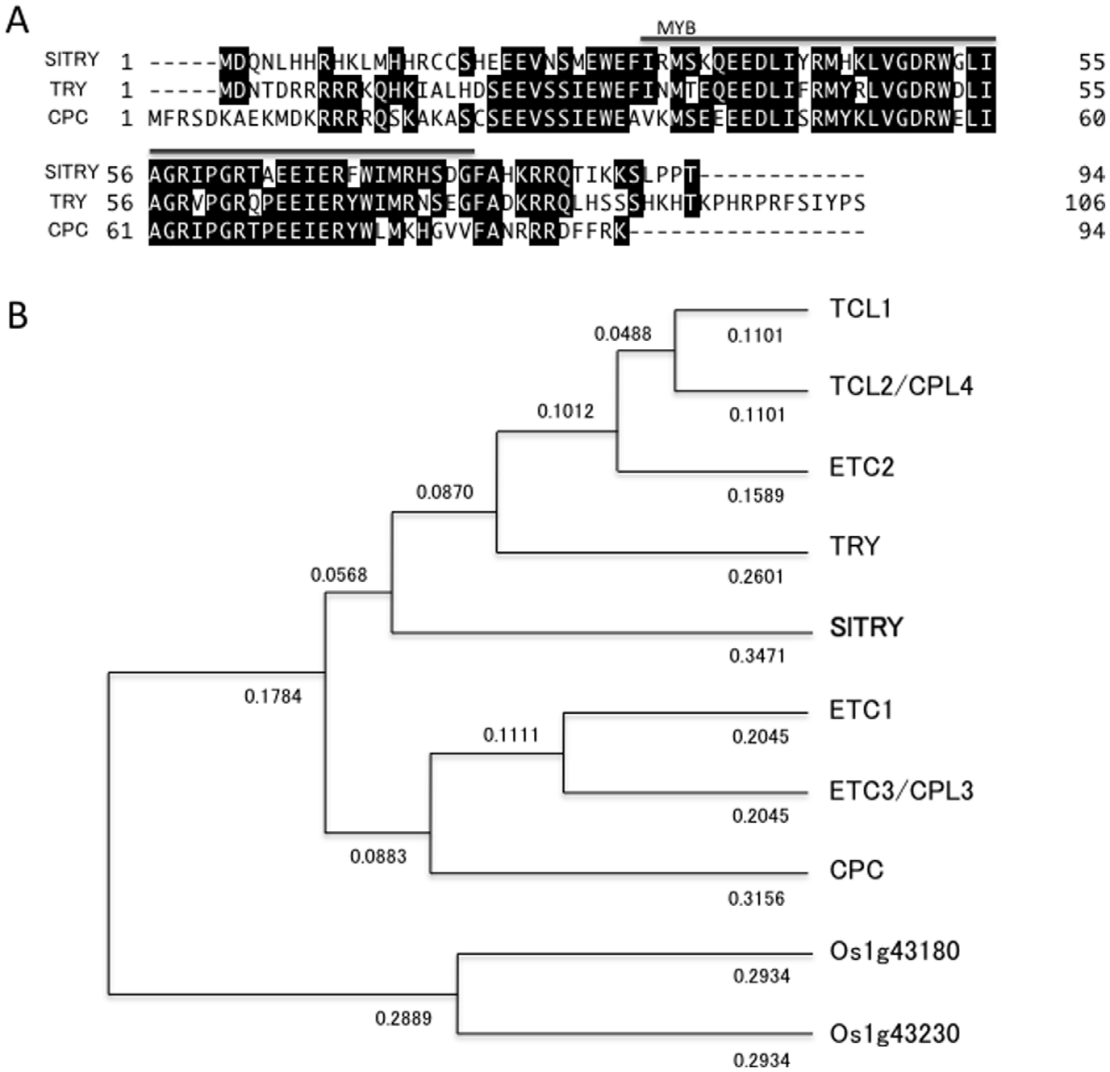
Amino acid sequence and phylogenic tree of CPC-like R3 MYB proteins. (A) Sequence alignment of SlTRY (Solyc01g095640.1.1), TRY (AC007288) and CPC (FJ268773). Shaded letters indicate identical residues. R3 MYB domains are indicated by a line above the sequences. (B) Phylogenic tree based on deduced amino acid sequences of CPC-like R3 MYB proteins [SlTRY, TRY, CPC, ETC1 (NM100020), ETC2 (FJ972652), ETC3/CPL3 (AB264292), TCL1 (FJ972675), TCL2/CPL4 (FJ972681), Os1g43180 and Os1g43230] were aligned with a multiple alignment program (Genetyx ver. 16.0.2 software, Genetyx, Tokyo, Japan), and a dendrogram was created using clustering with the Unweighted Pair Group Method with Arithmetic Mean (UPGMA). Branch length indicates relative evolutionary distances. Numbers above branches are genetic distances based on 10,000 bootstrap replicates. Distances are shown as the p-distance.

To find transcription factors regulating trichome and root-hair differentiation of tomato epidermis, we searched a tomato genome database (http://solgenomics.net/). We identified tomato homologs of the Arabidopsis *CPC* and *GL3* genes and named them *SlTRY* (Solyc01g095640.1.1) and *SlGL3* (Solyc08g081140.2.1), respectively. Members of the *CPC* family encode R3 type MYB transcription factor proteins in Arabidopsis [1]–[10]. The *SlTRY* encoded protein is more closely related to TRY than CPC (Figure 1A). Alignment of the amino acid sequences showed that the full length SlTRY protein shares 53% amino acid identity with TRY, and 50% with CPC. The R3 MYB motif of SlTRY shares 73% amino acid identity with that of TRY, and 71% with that of CPC.

To provide a framework for examining R3 MYB transcription factor evolution, we estimated the phylogeny of CPC-like R3 MYB transcription factor proteins from Arabidopsis (CPC, TRY, ETC1, ETC2, ETC3/CPL3, TCL1 and TCL2/CPL4), rice (*Oryza sativa*) (Os1g43180 and Os1g43230) and tomato (SlTRY) based on their deduced amino acid sequences (Figure 1B). SlTRY was more closely related to TRY than CPC, which belongs to a cluster that includes TCL1, TCL2/CPL4, ETC2, and TRY (Figure 1B). ETC1, ETC3/CPL3 and CPC belong to another cluster branching from the TRY subgroup. Consistent with previous reports, phylogenic analyses using the entire amino acid sequence showed that the CPC-like MYB family can be divided into two groups: TRY, ETC2, TCL1 and TCL2/CPL4 in one group and CPC, ETC1 and ETC3 in the other [6]–[9]. As previously described, the two rice orthologs, Os01g43180 and Os01g43230, form a distinct clade from the Arabidopsis CPC-like R3 MYB family (Figure 1B) [7].

**Figure 2:**
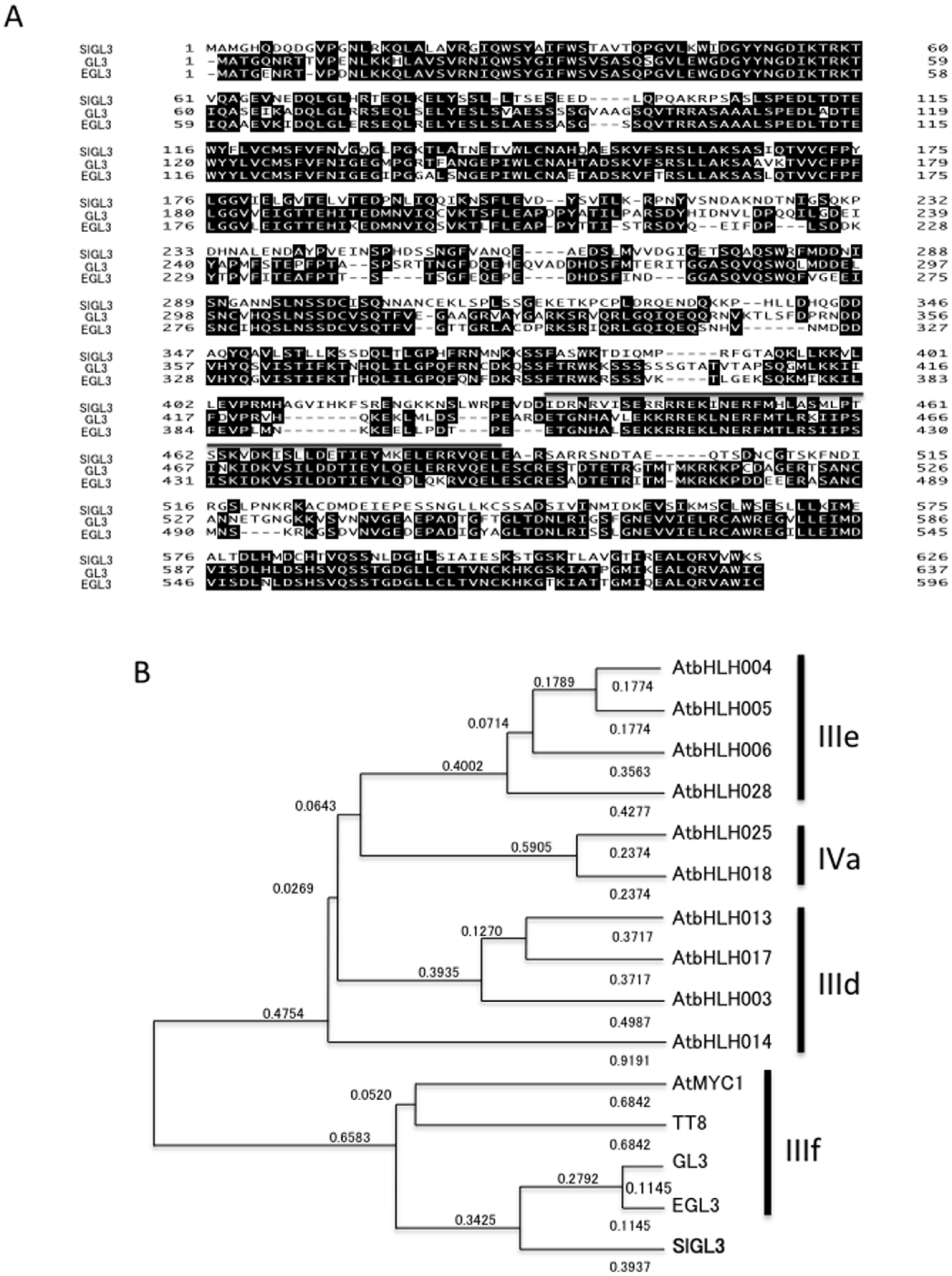
Amino acid sequence and phylogenic tree of bHLH proteins. (A) Sequence alignment of SlGL3 (Solyc08g081140.2.1), GL3 (AF246291) and EGL3 (NM20235). Shaded letters indicate identical residues. bHLH regions are indicated as line above the sequences. (B) Phylogenic tree based on deduced amino acid sequences of bHLH proteins [SlGL3, GL3, EGL3, TT8 (AJ277509), AtMYC1 (AF251697), AtbHLH003 (AF251688), AtbHLH004 (AF251689), AtbHLH005 (AF251690), AtbHLH006 (X99548), AtbHLH013 (AY120752), AtbHLH014 (AJ619812), AtbHLH017 (AY094399), AtbHLH018 (AF488562), AtbHLH025 (AF488567) and AtbHLH028 (AF252636)] aligned with a multiple alignment program (Genetyx ver. 16.0.2 software, Genetyx, Tokyo, Japan). The dendrogram was created using clustering with the Unweighted Pair Group Method with Arithmetic Mean (UPGMA). Branch length indicates relative evolutionary distances. Numbers above branches are genetic distances based on 10,000 bootstrap replicates. Distances are shown as the p-distance. Subdivision groups of Arabidopsis bHLH proteins (Group IIId, IIIe, IIIf and IVa) are shown to the right of the gene names.

The *SlGL3* encoded protein is closely related to the Arabidopsis bHLH transcription factor proteins encoded by *GL3* and *EGL3* (Figure 2A). Alignment of the amino acid sequences showed that the full length SlGL3 protein shares 45% amino acid identity with GL3, and 46% with EGL3. The bHLH motif of SlGL3 shares 46% amino acid identity with that of GL3, and 50% with that of EGL3. Based on the amino acid sequences of the bHLH regions, the Arabidopsis bHLH transcription factors are classified into 12 groups (I-XII) [50]. Group III contains 6 subgroups (IIIa-f), and GL3 and EGL3 belong to the IIIf subgroup [50].

To characterize SlGL3, we evaluated the phylogeny of bHLH transcription factor proteins (Figure 2B). Clustering in a phylogenic tree constructed from subgroups IIId (AtbHLH003, AtbHLH013, AtbHLH014 and AtbHLH017), IIIe (AtbHLH004, AtbHLH005, AtbHLH006 and AtbHLH028), IIIf (GL3, EGL3, TT8 and AtMYC1) and IVa (AtbHLH018, AtbHLH020 and AtbHLH025) was similar to the clustering in previously reported phylogenic trees [50]–[52] (Figure 2B). SlGL3 belongs to the IIIf subgroup and is more closely related to GL3 and EGL3 than AtMYC1 and TT8 (Figure 2B).

### Expression Patterns of the *SlTRY* and *SlGL3* Genes in Tomato

**Figure 3:**
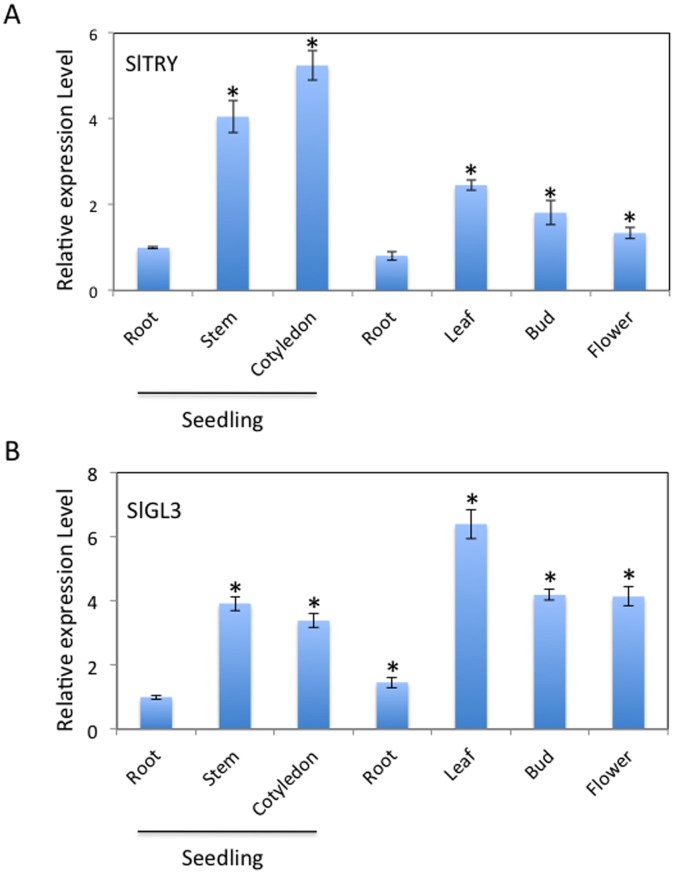
Tomato *SlTRY* and *SlGL3* gene expression. (A) Real-time reverse transcription PCR analysis of *SlTRY* gene expression in tomato organs. (B) Real-time reverse transcription PCR analysis of *SlGL3* gene expression in tomato organs. Total RNA was isolated from the indicated tissues from 7-day-old seedlings and 5-week-old plants. Expression levels of *SlTRY* and *SlGL3* in each organ relative to those in the seedling root were shown. The experiments were repeated three times. Error bars indicate the standard error. Bars marked with asterisks indicate a significant difference between the seedling root and the other organs by Student’s *t*-test (P<0.050).

Expression of *SlTRY* and *SlGL3* was examined in tomato tissues using real-time reverse transcription PCR. *SlTRY* was strongly expressed in stem and cotyledons of 7-day-old seedlings (Figure 3A). The relative expression level of *SlTRY* in cotyledon tissues was approximately 5 times greater than that in seedling root tissues (Figure 3A). The strongest expression of *SlGL3* was observed in 5-week-old plant leaves (Figure 3B). The relative expression level of *SlGL3* in true leaf tissues was approximately 6 times greater than that in seedling root tissues (Figure 3B). Both *SlTRY* and *SlGL3* were more strongly expressed in aerial tissues (including stem, cotyledon, leaf, bud and flower) than in roots. These results suggest that both *SlTRY* and *SlGL3* act in both shoot and root tissues and might have relatively strong functions in the aerial parts of plants.

### *SlTRY* Gene Functions in Trichome and Root-hair Development in Arabidopsis

**Figure 4:**
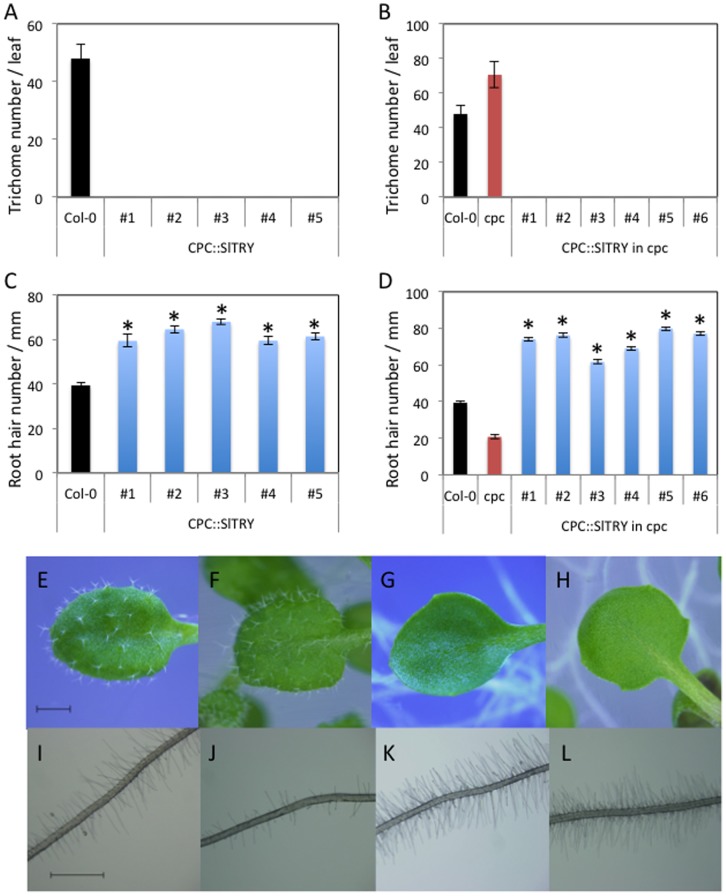
Trichome and root hair phenotypes of *CPC::SlTRY* transgenic plants. (A) Trichome formation on 2-week-old Arabidopsis third leaves of wild-type Col-0 and *CPC*::*SlTRY* (#1, #2, #3, #4 and #5). (B) Trichome formation on 2-week-old Arabidopsis third leaves of wild-type Col-0, *cpc-2* mutant and *CPC*::*SlTRY* in *cpc-2* (#1, #2, #3, #4 and #5). Number of trichomes per leaf was determined by counting a minimum of five 2-week-old third leaves from each line. (C) Root hair formation in 5-day-old Arabidopsis seedlings of wild-type Col-0 and *CPC*::*SlTRY* (#1, #2, #3, #4 and #5). (D) Root hair formation in 5-day-old Arabidopsis seedlings of wild-type Col-0, *cpc-2* mutant and *CPC*::*SlTRY* in *cpc-2* (#1, #2, #3, #4 and #5). The number of root hairs per mm was determined by counting a minimum of ten 5-day-old seedlings from each line. Error bars indicate the standard error. Bars marked with asterisks indicate a significant difference between the wild-type Col-0 and the transgenic lines (C), or the *CPC-2* mutant and the transgenic lines (D) by Student’s *t*-test (P<0.050). Trichome phenotypes of wild-type Col-0 (E), *cpc-2* (F), *CPC::SlTRY* (G) and *CPC::SlTRY* in *cpc-2* (H). Root hair phenotypes of wild-type Col-0 (I), *cpc-2* (J), *CPC::SlTRY* (K) and *CPC::SlTRY* in *cpc-2* (L). Scale bars: 1 mm.

To see if SlTRY is functionally similar to the CPC family of MYB transcription factors, we introduced *SlTRY* into Arabidopsis wild-type Col-0 plants under the control of the *CPC* promoter (*CPC::SlTRY*). The *CPC-like MYB* genes are thought to function redundantly in trichome and root-hair formation. For example, *35S::CPC, 35S::ETC1,* and *35S::ETC3* transgenic plants are all trichome-deficient and have a greater number of root-hairs [1], [3], [4], [7]. Consistent with these previous observations, all homozygous *CPC::SlTRY* transgenic lines (#1–#5) have the no-trichome phenotype, although wild-type Col-0 produces approximately 50 trichomes on the adaxial surface of the third true leaf (Figure 4A, E, G). To see a SlTRY function more clearly, we introduced *CPC::SlTRY* into the *cpc-2* mutant. As previously reported, the *cpc-2* mutant has a greater number of trichomes than wild-type [7] (Figure 4B, F). All homozygous *CPC::SlTRY* in *cpc-2* transgenic lines (#1–#6) show the no-trichome phenotype (Figure 4A, G) as observed in *CPC::SlTRY* in a wild-type background (Figure 4A). These results indicate that the tomato SlTRY protein has a function similar to the Arabidopsis CPC-like MYB proteins in regulating trichome development.

On the other hand, all homozygous *CPC::SlTRY* transgenic lines (#1–#6) produced greater numbers of root-hairs compared with wild-type Col-0, which produces approximately 40 root-hairs per mm (Figure 4C, I, K). This result is similar to the root-hair numbers of CPC-like MYB over-expressors [1], [3], [4], [7]. Homozygous *CPC::SLTRY* in *cpc-2* transgenic lines (#1–#6) also showed a greater number of root-hairs compared with wild-type and *cpc-2*, a mutant that produces a decreased number of root-hairs. These results are similar to the previously reported results using *CPC::CPC* in *cpc-2* or *GL2::CPC* in *cpc-1* transgenic plants [53], [54] (Figure 4D, J, L). These results indicate that the tomato protein SlTRY has a function similar to Arabidopsis CPC-like MYB proteins in regulating root-hair development. Together, our results show that SlTRY functions similar to CPC-like MYBs both in trichome and root-hair formation in Arabidopsis.

### *SIGL3* does not Function in Trichome and Root-hair Development in Arabidopsis

**Figure 5:**
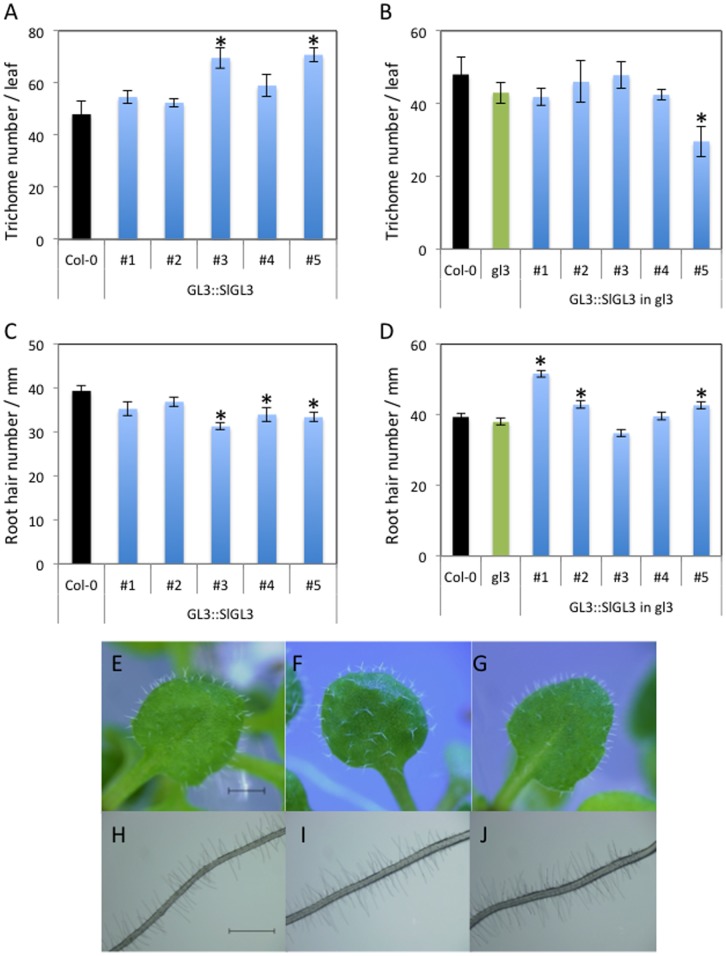
Trichome and root hair phenotypes of *GL3::SlGL3* transgenic plants. (A) Trichome formation on 2-week-old Arabidopsis third leaves of wild-type Col-0 and *GL3::SlGL3* (#1, #2, #3, #4 and #5). (B) Trichome formation on 2-week-old Arabidopsis third leaves of wild-type Col-0, *gl3-7454* mutant and *CPC*::*SlTRY* in *gl3-7454* (#1, #2, #3, #4 and #5). Number of trichomes per leaf was determined by counting a minimum of five 2-week-old third leaves from each line. (C) Root hair formation in 5-day-old Arabidopsis seedlings of wild-type Col-0 and *GL3::SlGL3* (#1, #2, #3, #4 and #5). (D) Root hair formation in 5-day-old Arabidopsis seedlings of wild-type Col-0, *gl3-7454* mutant and *CPC*::*SlTRY* in *gl3-7454* (#1, #2, #3, #4 and #5). The number of root hairs per mm was determined by counting a minimum of ten 5-day-old seedlings from each line. Error bars indicate the standard error. Bars marked with asterisks indicate a significant difference between the wild-type Col-0 and the transgenic lines [(A), (C)], or the *gl3-7454* mutant and the transgenic lines [(B), (D)] by Student’s *t*-test (P<0.050). Trichome phenotypes of *gl3-7454* (E), *GL3::SlGL3* (F), and *GL3::SlGL3* in *gl3-7454* (G). Root hair phenotypes of *gl3-7454* (H), *GL3::SlGL3* (I), and *GL3::SlGL3* in *gl3-7454* (J). Scale bars: 1 mm.

To see if SlGL3 is functionally similar to Arabidopsis GL3 or EGL3, we introduced *SlGL3* into Arabidopsis wild-type Col-0 under the control of the *GL3* promoter (*GL3::SlGL3*). The *GL3* and *EGL3* genes are thought to be involved in trichome and root-hair formation. *GL3* or *EGL3* over-expressors produce greater numbers of trichomes and reduced numbers of root-hairs [12], [13]. Most of homozygous *GL3::SlGL3* transgenic lines produced the similar number of trichomes as that of wild-type (Figure 5A, F). Unlike the previous observation from complementation analysis of the *gl3-1* mutant by *GL3::GL3* [12], homozygous *GL3::SlGL3* in *gl3-7454* transgenic plants did not have an increased number of trichomes compared with the *gl3-7454* mutant (Figure 5B, E, G). On the contrary, one of the *GL3::SlGL3* in *gl3-7454* transgenic lines (#5) had significantly fewer trichomes in comparison with the *gl3-7454* mutant (Figure 5B). These results suggest that tomato *SlGL3* may not have a function same to Arabidopsis *GL3*.

In addition to the effects on trichome number, GL3 is known to affect the trichome branching phenotype [12]. As observed in the *gl3-1* mutant (Ler background) [12], trichomes of the *gl3-7454* mutant (Col-0 background) had fewer branches than wild-type Col-0 (Table 2). Introduction of the *GL3::SlGL3* gene did not rescue the decreased branch number phenotype of the *gl3-7454* mutant trichomes (Table 2). This result suggests that the *SlGL3* gene does not have a function similar to *GL3* in the induction of trichome branching.

**Table 2:** Trichome branch numbers.

	branches (br)/trichome (%)			
Genotype	1 br	2 br	3 br	4 br
Col-0	0	12±2	86±4	2±1
*gl3*	26±9	71±10	3±2	0
*GL3::SlGL3*	1±1	40±14	57±13	2±1
*GL3::SlGL3* in *gl3*	30±13	64±12	6±4	0

Data, including s.d., were obtained from at least 10 two-week-old third leaves from each line.

Three of five homozygous *GL3::SlGL3* transgenic lines (#3–#5) produced significantly fewer root-hairs compared with wild-type (Figure 5C, I). This result is similar to the tendency for fewer root-hairs in the *GL3* or *EGL3* over-expressors [13], but the effect of *SlGL3* was weaker than that of *GL3* and *EGL3*. In contrast, three of five homozygous *GL3::SlGL3* plants in *gl3-7454* transgenic lines (#3–#5) produced significantly higher numbers of root-hairs compared with wild-type Col-0 and *gl3-7454* (Figure 5D, H, J).

### Expression of the *GL2* Gene in *SlTRY* expressing Plants

**Figure 6:**
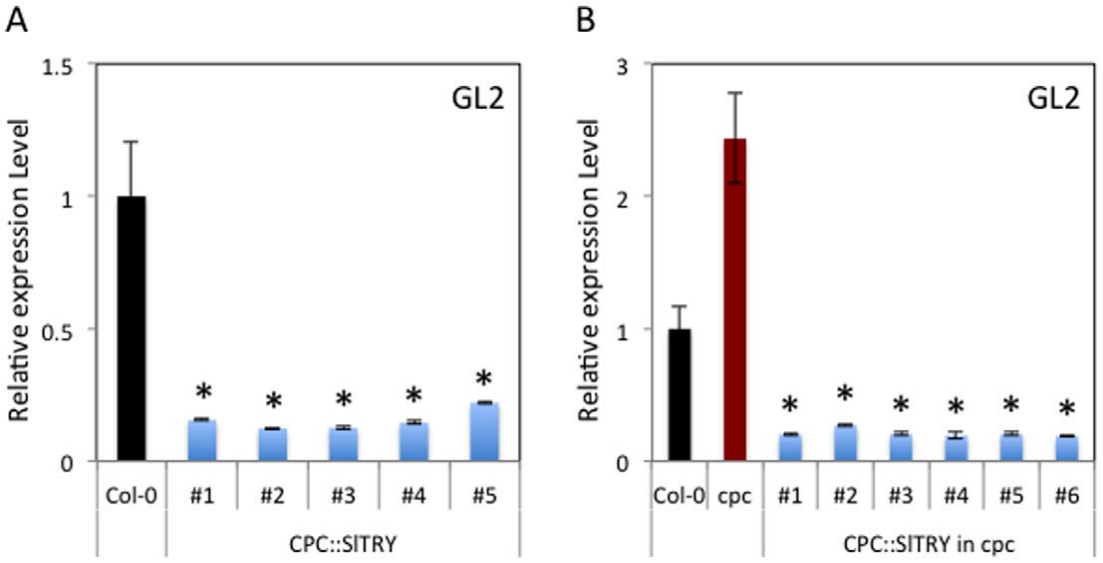
*GL2* expression in the *CPC::SlTRY* transgenic plants. Real-time reverse transcription PCR analyses of the *GL2* gene in wild-type Col-0 and *CPC*::*SlTRY* (#1, #2, #3, #4 and #5) (A), and wild-type Col-0, *cpc-2* mutant and *CPC*::*SlTRY* in *cpc-2* (#1, #2, #3, #4 and #5) (B). Expression levels were normalized to *Act2* expression. An expression level of *GL2* in each line relative to that in wild-type was indicated. The experiments were repeated three times. Error bars indicate the standard error. Bars marked with asterisks indicate a significant difference between the wild-type Col-0 and the transgenic lines (A), or the *cpc-2* mutant and the transgenic lines (B) by Student’s *t*-test (P<0.050).

To determine whether *CPC::SlTRY* functions (Figure 4) were due to epistatic effects of *SlTRY* on *GL2* activity, we carried out real-time reverse transcription PCR analyses using *GL2* primers (Figure 6). The *GL2* gene is thought to act downstream of the MYB-bHLH transcriptional complex to promote trichome formation and inhibit root-hair formation [1], [14]–[17]. Consistent with the *CPC::SlTRY* transgene phenotype (Figure 4A), *GL2* expression was strongly repressed in all *CPC::SlTRY* transgenic lines (Figure 6A). In the *CPC::SlTRY* in *cpc-2* transgenic lines, *GL2* expression was also strongly repressed compared with wild-type and *cpc-2* as was the case in the wild-type background (Figure 6B). To compare gene expression levels of the introduced gene among transgenic lines, we checked GFP expression since GFP was fused to the C-terminal region of SlTRY (Figure S1A). Although the transgene expression levels varied depending on the lines (Figure S1A), expression in all lines was strong enough to repress *GL2* expression.

### Expression of the *GL2* Gene in *SlGL3* expressing Plants

**Figure 7:**
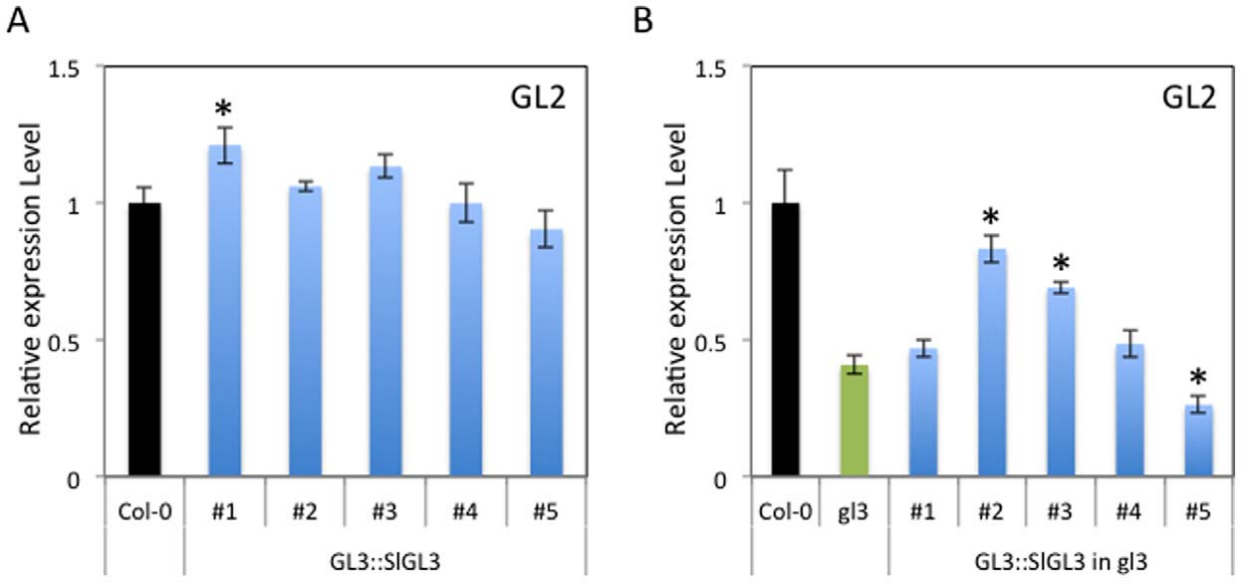
*GL2* expression in *CPC::SlGL3* transgenic plants. Real-time reverse transcription PCR analyses of the *GL2* gene in wild-type Col-0 and *CPC*::*SlGL3* (#1, #2, #3, #4 and #5) (A), and wild-type Col-0, *gl3-7454* mutant and *CPC*::*SlGL3* in *gl3-7454* (#1, #2, #3, #4 and #5) (B). Expression levels were normalized to *Act2* expression. An expression level of *GL2* in each line relative to that in wild-type was indicated. The experiments were repeated three times. Error bars indicate the standard error. Bars marked with asterisks indicate a significant difference between the wild-type Col-0 and the transgenic lines (A), or the *gl3-7454* mutant and the transgenic lines (B) by Student’s *t*-test (P<0.050).

To determine whether *CPC::SlGL3* functions (Figure 5) were due to epistatic effects of *SlGL3* on *GL2* activity, we also carried out real-time reverse transcription PCR analyses using *GL2* primers (Figure 7). Inconsistent with the *GL3::SlGL3* transgene phenotypes (Figure 5A), significant *GL2* expression changes were observed only in *GL3::SlGL3* line #1 compared with wild-type Col-0 (Figure 7A). Apparently, *SlGL3* does not have a remarkable effect on *GL2* expression. In *GL3::SlGL3* in *gl3-7454* transgenic plants, a significant increase in *GL2* expression was observed in lines #2 and #3 compared with that in the *gl3-7454* mutant; however, these *GL2* expression levels did not reach similar expression levels of *GL2* in wild-type Col-0 (Figure 7B). A significant decrease in *GL2* expression was observed in line #5 compared with that in the *gl3-7454* mutant (Figure 7B). Thus, we checked *GFP* expression that should reflect the introduced *SlGL3* expression levels since the *SlGL3* construct was fused to *GFP* (Figure S1B). The *GFP* expressions varied greatly among *GL3::SlGL3* in *gl3-7454* transgenic lines (Figure S1B). In addition, the relative expression levels of *GFP* in *GL3::SlGL3* in *gl3-7454* lines were lower than that in the *GL3::SlGL3* lines (Figure S1B). These results suggest that *SlGL3* expression was unstable in the *gl3-7454* mutant background.

## Discussion

In this study, we identified tomato *SlTRY* and *SlGL3* genes that were orthologous to the Arabidopsis *TRY* and *GL3* genes, respectively. Recently, a high-quality genome sequence of tomato was released by the Tomato Genome Consortium [55]. Since tomatoes are very distantly related to Arabidopsis evolutionarily, these sequence data may offer important information about plant evolution in the future. The functional analyses of the *SlTRY* and *SlGL3* genes in this study provide insights into tomato trichome and root-hair evolution. Branching of the SlTRY and CPC clusters from a common trunk in the phylogenic tree suggests that the evolution of tomato and Arabidopsis CPC-like R3 MYB genes began with duplication of a single common ancestor after divergence from rice (Figure 1B). Based on the functions of known members of the Arabidopsis bHLH transcription factor family, it was hypothesized that different members participate in distinct developmental processes [50]. Among the genes, members of the IIIf subgroup, including AtMYC1, TT8, GL3 and EGL3 function in trichome and root-hair development, flavonoid/anthocyanin metabolism, and/or mucilage biosynthesis [13], [19], [20], [56]–[59]. Phylogenic analyses predicted that tomato SlGL3 evolved from a common ancestor to Arabidopsis GL3 and EGL3 after divergence of the IIIf subgroup from other bHLH subgroups (Figure 2B). Thus, we expected that SlTRY and SlGL3 would have similar functions to TRY/CPC and GL3/EGL3 in trichome and root-hair differentiation. Both *SlTRY* and *SlGL3* were shown to be expressed in all the tissues examined, especially in the aerial parts (Figure 3), suggesting that these genes function in nearly the entire tomato plant body.

In our experiment, *SlTRY* was demonstrated to function quite similarly to the CPC-like MYB transcription factors in Arabidopsis trichome and root-hair formation (Figure 4). Both *CPC::SlTRY* and *CPC::SlTRY* in *cpc-2* transgenic plants showed the no-trichome and increased root-hair phenotypes (Figure 4). Previously, an R3-type MYB of TRY and an R2R3-type MYB of GL1 were reported to compete for a GL3 binding site to form different types of MYB-bHLH complexes involved in Arabidopsis trichome differentiation [60]. TRY prevents the interaction between GL1 and GL3 [19], and CPC physically interacts with GL3/EGL3 [13], suggesting a competition model for CPC and WER [13], [16]. The CPC protein was proposed to disrupt the WER-GL3/EGL3 protein complex by competitive binding with WER, leading to repression of *GL2* expression [18], [53], [54]. In this study, we showed that SlTRY also repressed *GL2* expression (Figure 6). These results suggest that the SlTRY protein may also disrupt the MYB-bHLH complex of GL1/WER-GL3/EGL3, leading to repression of *GL2* expression.

In contrast to SlTRY, SlGL3 did not show clear GL3/EGL3-like functions for trichome and root-hair differentiation in Arabidopsis (Figure 5). Overexpression of GL3 and/or EGL3 induced a notable increase in trichome number and a decrease in root-hair number in Arabidopsis [12], [13]. However, in our experiment, only two of five and three of five *GL3::SlGL3* transgenic lines showed a significant increase in trichome number and a significant decrease in root-hair number compared with wild-type, respectively (Figure 5A and B). It is thus possible that tomato SlGL3 has an evolutionarily conserved, highly homologous amino acid sequence and only a partially similar function to Arabidopsis GL3/EGL3. Since trichome and root-hair structure differs between Arabidopsis and tomato, the *SlGL3* gene may have acquired another function from *GL3/EGL3* during evolution. The functional difference between SlGL3 and GL3/EGL3 may be derived from the relatively low amino acid homology region in the bHLH motifs (Figure 2A). Only one of five *GL3::SlGL3* transgenic lines showed a significant increase in the *GL2* expression level compared with wild-type Col-0 (Figure 7A). Thus, two possibilities exist. First, a low affinity of SlGL3 protein to WER/GL1 proteins may result in the formation of an incomplete MYB-bHLH protein complexthat cannot activate *GL2* expression. Recently, Zhao et al. reported that a single amino acid substitution in another *GL3* homologous gene *AtMYC1,* leads to trichome and root-hair patterning defects by abolishing its interaction with partner proteins in Arabidopsis [61]. Arginine (R173) in the AtMYC1 protein is an essential amino acid residue for interaction with MYB proteins for proper functions [61]. We confirmed that there is a conserved Arg in the SlGL3 protein as in the GL3, EGL3 and AtMYC1 proteins (Figure 2A). Thus, some amino acid substitution other than Arg may contribute to the functional difference between SlGL3 and GL3/EGL3. Second, SlGL3 might have lost either the DNA binding ability to the *GL2* promoter region or the ability to activate the *GL2* promoter. For example, we previously reported that WER loses its DNA binding ability by at least two amino acid substitutions [53]. Tomato SlGL3 may have lost its DNA binding ability to the *GL2* promoter region during evolution.

In order to compare our results in the same Col-0 background, we used the *gl3-7454* mutant for the complementary experiment. The *gl3-7454* mutant shows only a mild phenotype compared with the *gl3-1* mutant (Ler:Landsberg erecta background) [12]. The *gl3-7454* mutant shows no significant difference in trichome number or in root-hair number compared with wild-type Col-0 (Figure 5B and D). Unexpectedly, one of five *GL3::SlGL3* in *gl3-7454* transgenic lines showed significant decreases in trichome number compared with *gl3-7454* (Figure 5B), and three of five *GL3::SlGL3* in *gl3-7454* transgenic lines showed significant increases in root-hair number compared with *gl3-7454* (Figure 5D). Consistent with these unexpected phenotypes of *GL3::SlGL3* in *gl3-7454* transgenic plants, the relative expression levels of *GL2* varied (Figure 7B). Two of five *GL3::SlGL3* in *gl3-7454* transgenic lines showed significantly higher *GL2* expression levels compared with that in *gl3-7454*, but the level did not reach that in the wild-type Col-0 (Figure 7B). One of five *GL3::SlGL3* in *gl3-7454* transgenic lines showed significantly lower *GL2* expression levels compared with that in the *gl3-7454* mutant (Figure 7B). As checked by fusion of *SlGL3* to *GFP*, expression of introduced *SlGL3* was unstable and fluctuated depending on the lines (Figure S1). In addition, *SlGL3* did not rescue the reduced number of trichome branches phenotype of *gl3-7454* (Table 2). These data strongly suggest the functional divergence between tomato *SlGL3* and Arabidopsis *GL3/EGL3*. There are 158 bHLH genes in Arabidopsis [52], [62]. Tomato should have the similar or more number of the bHLH genes when the full annotations of tomato genes are determined. We concluded that there is other *GL3* ortholog(s) in the unannotated tomato genomes or tomato uses other pathways to regulate the epidermal cell differentiation.Additional investigations to further determine the functions of R3-MYB and bHLH in trichome and root-hair differentiation in tomato are necessary.

## Supporting Information

Figure S1 ***GFP*** **expression in the transgenic plants.** Real-time reverse transcription PCR analyses of the *GFP* gene in *CPC*::*SlTRY* (#1, #2, #3, #4 and #5) (A), *CPC*::*SlTRY* in *cpc-2* (#1, #2, #3, #4 and #5) (B), *CPC*::*SlGL3* (#1, #2, #3, #4 and #5) (C), and *CPC*::*SlGL3* in *gl3-7454* (#1, #2, #3, #4 and #5) (D). Expression levels were normalized to *Act2* expression. Relative expression levels: expression levels of *GFP* in each line relative to each transgenic line #1. The experiment was repeated three times. Error bars indicate the standard error.
(TIFF)
