# Supplementary figures and images for: Control of Plant Trichome and Root-Hair Development by a Tomato (Solanum lycopersicum) R3 MYB Transcription Factor
Source: PLoS One. 2013 Jan 11;8(1):e54019. doi: 10.1371/journal.pone.0054019 (PMC3543402; doi:10.1371/journal.pone.0054019)

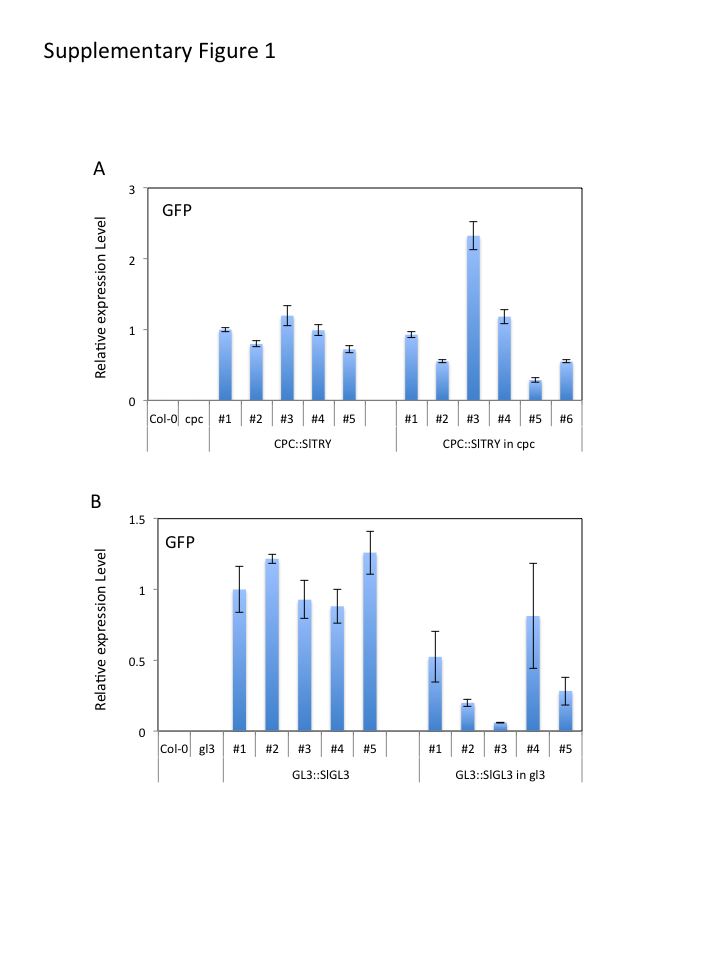

Supplement: Figure S1 — GFP expression in the transgenic plants. Real-time reverse transcription PCR analyses of the GFP gene in CPC::SlTRY (#1, #2, #3, #4 and #5) (A), CPC::SlTRY in cpc-2 (#1, #2, #3, #4 and #5) (B), CPC::SlGL3 (#1, #2, #3, #4 and #5) (C), and CPC::SlGL3 in gl3-7454 (#1, #2, #3, #4 and #5) (D). Expression levels were normalized to Act2 expression. Relative expression levels: expression levels of GFP in each line relative to each transgenic line #1. The experiment was repeated three times. Error bars indicate the standard error. (TIFF) [file pone.0054019.s001.tiff]
